# Intriguing insight into unanswered questions about Mpox: exploring health policy implications and considerations

**DOI:** 10.1186/s12961-024-01123-9

**Published:** 2024-03-22

**Authors:** Emery Manirambona, Sheharyar Hassan Khan, Abdelmonem Siddiq, Khaled Albakri, Hazem Mohamed Salamah, Noheir Ashraf Ibrahem Fathy Hassan, Shuaibu Saidu Musa, Kuldeep Dhama

**Affiliations:** 1 College of Medicine and Health Sciences, University of Rwanda, Kigali, Rwanda; 2 King Edward Medical University, Lahore, Pakistan; 3 Faculty of Pharmacy, Mansoura University, Mansoura, 35516 Egypt; 4 Faculty of Medicine, The Hashemite University, Zarqa, Jordan; 5 Faculty of Medicine, Zagazig University, Zagazig, Egypt; 6 Faculty of Medicine, Aswan University, Aswan, Egypt; 7 Department of Nursing Science, Ahmadu Bello University, Zaria, Nigeria; 8 Division of Pathology, ICAR-Indian Veterinary Research Institute (IVRI), Izatnagar, Bareilly, Uttar Pradesh 243122 India

**Keywords:** Monkeypox, Mpox, Mysteries, Unanswered questions, Vaccines, Treatment, Prevention controls, Health Policy

## Abstract

The 2022 multi-country Monkeypox (Mpox) outbreak has added concerns to scientific research. However, unanswered questions about the disease remain. These unanswered questions lie in different aspects, such as transmission, the affected community, clinical presentations, infection and prevention control and treatment and vaccination. It is imperative to address these issues to stop the spread and transmission of disease. We documented unanswered questions with Mpox and offered suggestions that could help put health policy into practice. One of those questions is why gay, bisexual or other men who have sex with men (gbMSM) are the most affected community, underscoring the importance of prioritizing this community regarding treatment, vaccination and post-exposure prophylaxis. In addition, destigmatizing gbMSM and implementing community-based gbMSM consultation and action alongside ethical surveillance can facilitate other preventive measures such as ring vaccination to curb disease transmission and track vaccine efficacy. Relevant to that, vaccine and drug side effects have implied the questionability of their use and stimulated the importance of health policy development regarding expanded access and off-label use, expressing the need for safe drug and vaccine development manufacturing. The possibility of reverse zoonotic has also been raised, thus indicating the requirement to screen not only humans, but also their related animals to understand the real magnitude of reverse zoonosis and its potential risks. Implementing infection prevention and control measures to stop the virus circulation at the human–animal interface that includes One Health approach is essential.

## Introduction

The 2022 global Monkeypox (Mpox) outbreak, caused by the monkeypox virus (MPXV), has emerged and spread unusually outside the endemic regions of Central and West Africa. The virus has affected 93 479 people around the globe and caused 177 deaths as of 7 February 2024 [1]. These figures raised the alarm for the international authorities after the first cluster of cases of the 2022 outbreak was discovered in the United Kingdom in May 2022 [2]. That alarm led the WHO to declare the 2022 global Mpox outbreak as a public health emergency of international concern (PHEIC) on 23 July 2022, which underlined the impact of the disease [3].

**Fig. 1:**
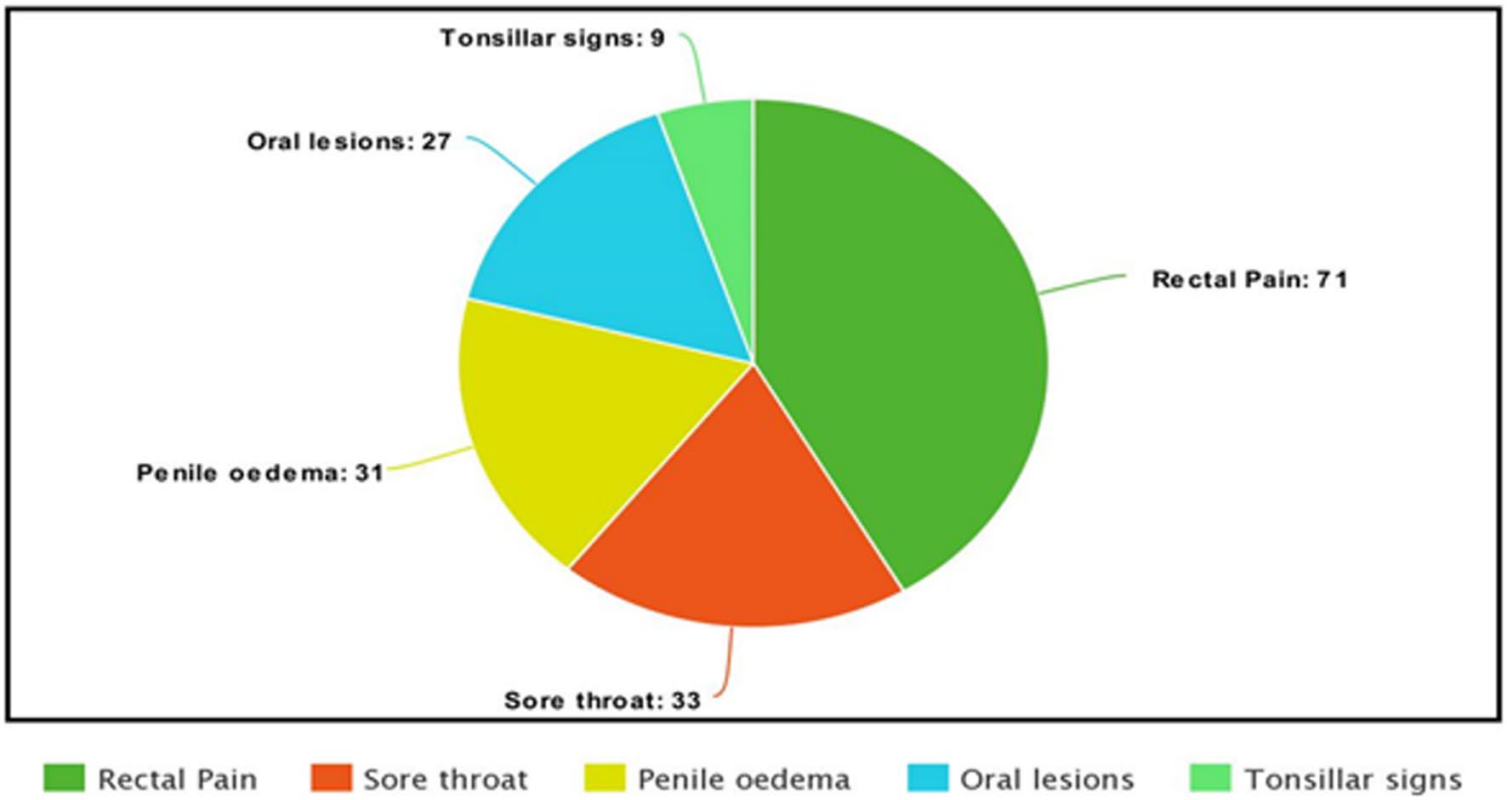
Number of patients with novel or atypical signs of Mpox (Source: Clinical features and unique presentations of human monkeypox in a central London centre during the 2022 outbreak: descriptive case series)

The 2022 Mpox outbreak has had a multicounty spread. Mpox cases have been more commonly identified among gay, bisexual or other men who have sex with men (gbMSM) outside endemic regions [4, 5]. In addition to this epidemiological trend, this group has shown unusual clinical presentations that include penile oedema, penile secondary infections, rectal problems ranging from simple rectal pain to rectal perforation and solitary lesions; features that could be mistaken for other pathologies such as syphilis. The presence of such symptoms in gbMSM raises the possibility of the sexual mode of transmission for the virus, which was not observed previously [5]. The new patterns of Mpox are highlighted in Fig. 1.

As mentioned above, the 2022 outbreak recorded a higher number of people affected worldwide than in the previous outbreaks documented in Sub-Saharan Africa, thus raising questions about the origin of the virus. Evidence showed that the possible reason behind the high virulence of the current Mpox outbreak was the viral evolution, and this was particularly revealed in a phylogenic study carried out in Portugal at its national laboratory reference [6]. In total, 136 genomes from African countries, the USA, the United Kingdom and Australia between the 1960s and 2022 that contained clades I, II and III were analysed in this phylogenetic analysis comparing MPXV viral sequences linked to the global outbreak of 2022. On the basis of MPXV global phylogeny, it was discovered that the 2022 outbreak cluster (lineage B.1) belonged to clade III [6]. This evidence also showed that the virus has undergone rapid evolution during its human-to-human transmission, which resulted in an increased level of transmissibility and more cases as a result. High virulence distinguishes Mpox clade III from the previous clades, owing to the repeated evolutionary changes that resulted in improved attack rate, widespread transmission, increased cases and atypical clinical findings, which need to be explored and addressed appropriately. To this aim, this study critically reviews questions to be addressed conclusively about Monkeypox for a better understanding of transmission and salient advances in vaccines, therapeutics and prevention control strategies to combat this global public health emergency. The data provided in this study will be useful for health policymakers to improve health systems preparedness and response to future outbreaks.

## Mpox transmission

Mpox transmission in the 2022 outbreak has been unusual compared with the Mpox outbreaks documented a few years before. First, the 2022 multi-country outbreak of Mpox has raised questions about the transmission of the virus, as clusters of cases were documented around the globe and without clear links to endemic countries. Eating contaminated animal meat has been identified as an additional potential method of disease transmission, in addition to animal-to-human transfers that could infect humans through direct contact or exposure to diseased animals, exudates from mucosal or cutaneous lesions, saliva and respiratory secretions [7]. Further data have suggested sexual intercourse as a potential source of infection following a significant number of infected people through sexual contact transmission [5, 8]. This is also linked to the fact that just more than 75% of infected persons declared in the United Kingdom were identified as gbMSM, making them the most vulnerable population to Mpox [7]. This raised the question of why this community is the most affected and what the factors associated with the predominance of the disease among gbMSM are. These multiple routes of virus transmission, atypical and unusual clinical disease presentations and asymptomatic infections uniquely seen during the 2022 Mpox outbreaks might lead to undetected cases and silent spread in humans [9]. This can be intensified by the probability of fomite transmission described in the case of contact with viral particles [7]. These viral particles can persist for up to 15 days on porous surfaces, bedding, clothing, towels, objects and electronics after being left by infected individuals [10], thus raising concerns on how to prevent fomite transmission mode. Interestingly, another study published in 2023 evaluating the stability of the Mpox virus revealed that the virus can survive for up to 30 days under certain temperatures, indicating the potential for reactivation and retransmission of the virus when favourable conditions appear [11]. This also raises the question of timely appropriate and effective prevention strategies.

Mpox cases have been identified among healthcare workers as a result of direct contact with affected individuals in healthcare settings or with the community at risk [12]. It has been documented that healthcare workers can be affected due to some strains of the virus that can develop resistance and survive in the healthcare environment, demonstrating again the possibility of fomite transmission [12]. On this basis, healthcare providers should be highly cautious as they are also in close and long-standing contact with the confirmed and suspected cases, making them among the groups with high vulnerability to infection.

Furthermore, congenital Mpox, which is the transmission of the virus from the mother to the fetus, has been revealed in previous outbreaks in the Democratic Republic of Congo, but was not reported in the outbreak starting in 2022 [13]. Similarly, whereas there is no suggestive evidence about the possibility of Mpox transmission through breast milk, breastfeeding women could transmit the virus through skin-to-skin contact [14].

It has also been suggested that Mpox is a zoonotic illness. Dogs close to positive human cases were the infected animals in certain cases of sick animals from Brazil and France, suggesting the possibility of a reverse zoonotic disease [15]. Enhancing screening of humans and animals in close contact is required to know the real magnitude of reverse zoonosis and its potential risks along with the implementation of needful infection prevention and control measures to limit virus circulation at the human–animal interface, including the One Health approach [16].

## Therapies and vaccines

As drugs play an essential role in treating infectious diseases, advances have been made in designing and developing effective prophylactic and therapeutic options to counter Mpox.

For instance, an antiviral drug, the tecovirimat 200 mg injection used in severe cases, was approved by the US Food and Drug Administration (FDA) on 22 February 2023 with a shelf life between 24 months and 42 months, just a month before authorizing its emergency use for the Cue Mpox molecular test in care settings [17, 18]. Another drug that has been used to treat Mpox is cidofovir, which has been effective when tested in vitro with promising results [19]. Although not regulated by any regulatory body to treat Mpox among humans, but licenced in the United Kingdom and by the FDA for cytomegalovirus retinitis, the use in intravenous cidofovir dose in the United Kingdom (5 m/kg) and in other cases of individuals infected with Mpox elsewhere has shown a significant clinical improvement, as revealed by the data published in *The Lancet* in 2023 [19].

Although the above-mentioned drugs are being used, they are still under trial. This illustrates the need for clinical surveillance and prohibition of off-label use without medical guidance. In connection with that, these medications have been authorized via a process known as expanded access, also known as compassionate use, which is used when a serious illness develops and no competent organ has authorized a safe and effective medication, allowing for the use of an experimental medication [17]. The potential patient benefits of expanded access support the use of the drug. However, risks can be significant among patients. Whereas there were mild side effects in the use of both of those drugs, major adverse effects such as nephrotoxicity, neutropenia and decreased ocular pressure were revealed in the case of cidofovir [20]. Those side effects imply the questionability of the use of the above drugs, stimulate the importance of health policy development regarding expanded access and express the need for safe drug development.

**Fig. 2:**
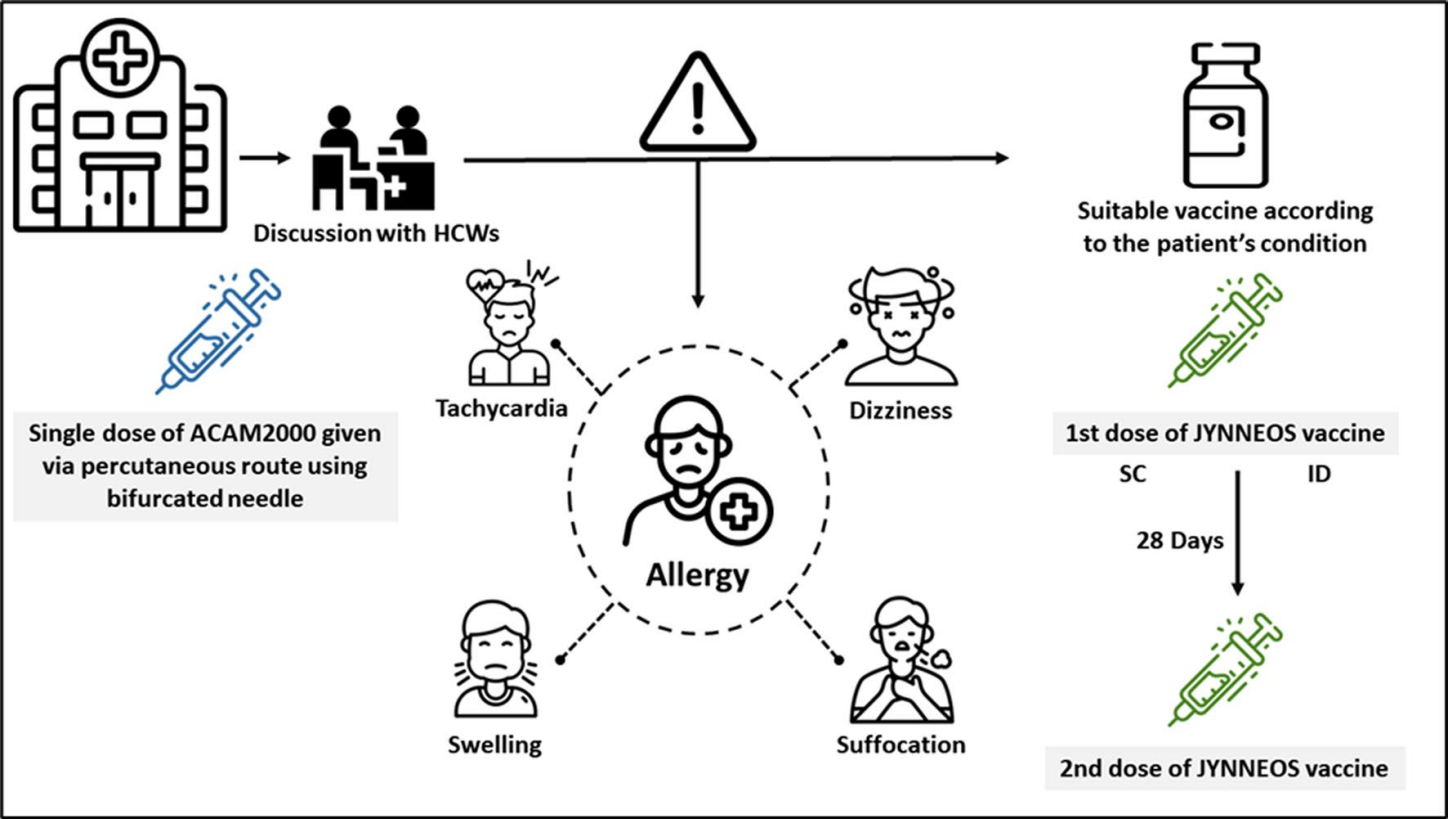
Informed consent between healthcare workers and individuals for getting the suitable vaccine, either JYNNEOS or ACAM2000, with the warning that the patient must be a patient in the hospital for a reasonable time for clinical surveillance, as there is a risk of developing severe allergic manifestations such as suffocation, swelling, tachycardia or dizziness, which are life-threatening conditions Sources: Highlights of prescribing information: JYNNEOS (Smallpox and Monkeypox Vaccine, Live, Nonreplicating), ACAM2000 [Smallpox (Vaccinia) Vaccine, Live]

JYNNEOS, an approved vaccine in 2019 by the FDA for smallpox and Mpox disease, is the only approved FDA vaccine for Mpox prevention, and is administrated in two subcutaneous injections 28 days apart. This vaccine has proven to be effective among vaccinated people who showed a lower incidence of Mpox than unvaccinated people [21]. However, questions remain regarding the level of protection after only one dose, as well as the duration of efficacy or the time the vaccinated person cannot again contract the disease. This is also shown in the fact that vaccinated people are required to keep minimizing the risk of transmission even in the post-vaccination period. In addition, JYNNEOS can have adverse effects that include fatigue, nausea, headache, muscle pain, chills, fever, and redness and swelling at the injection site as a systemic manifestation in addition to the local side effects such as pain, oedema, pruritis and induration [22, 23] (Fig. 2). The above-mentioned adverse effects demonstrate the importance of further improvement of the JYNNEOS vaccine.

Alternatively, ACAM2000 is a live virus replication competent that can prevent Mpox as a second choice after JYNNEOS. However, this attenuated strain of the vaccinia virus can result in serious complications, such as progressive vaccinia, particularly in persons with immunocompromised immune systems. Therefore, ACAM2000 has been contraindicated for persons living with human immunodeficiency virus (HIV) owing to being at a high risk of the spread of the vaccinia virus. The possible side effects show the importance of the informative discussion between the patient and the healthcare provider to gain informed consent before vaccine administration [24] (Fig. 2). The principle of informed consent is critical, as it ensures the preservation of the patient’s autonomy and makes the patient aware of possible advantages and risks of the suggested treatment necessary to make a well-informed decision.

**Fig. 3:**
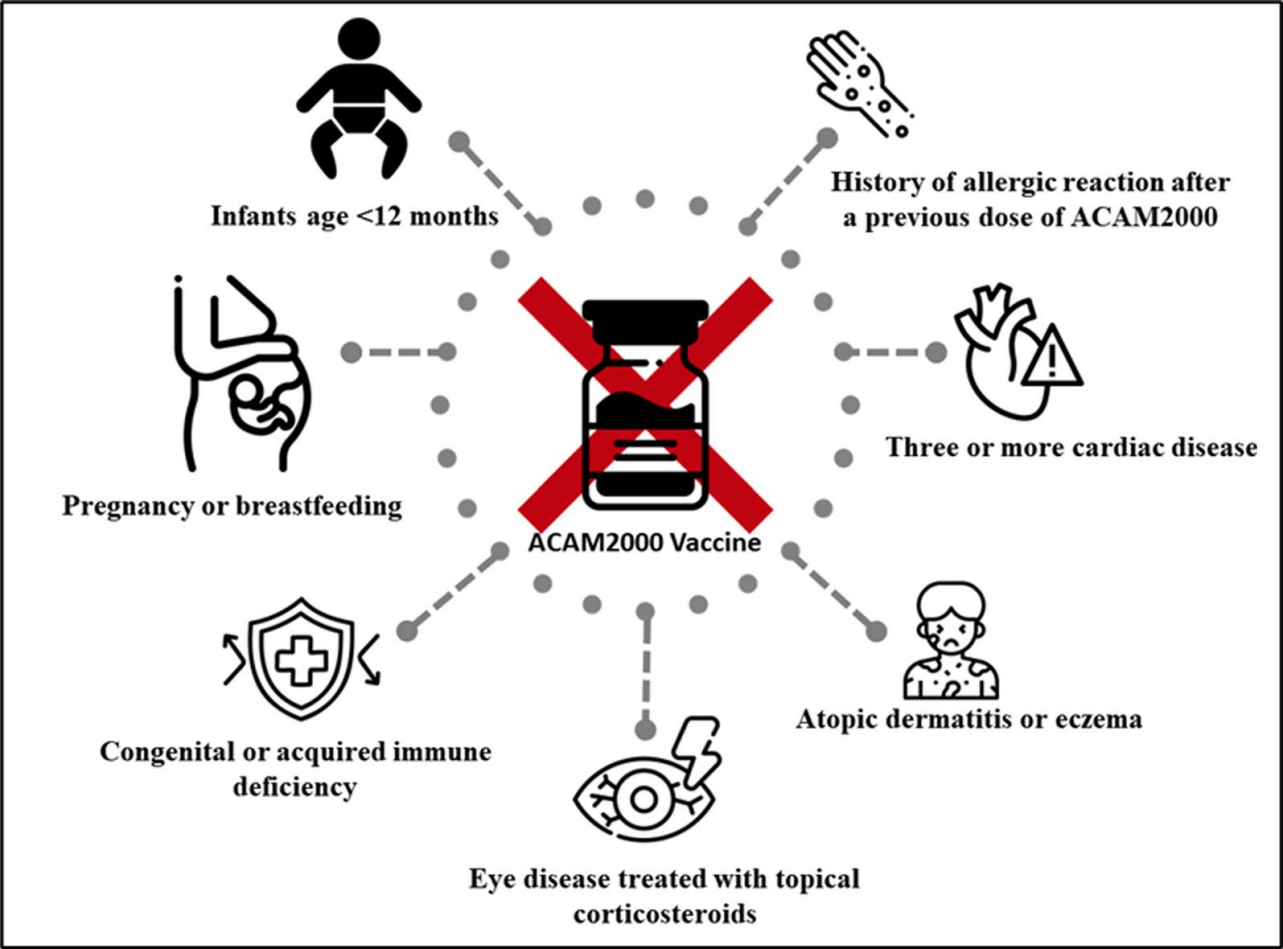
Contraindication for the ACAM2000 vaccine Source: Use of JYNNEOS (Smallpox and Monkeypox Vaccine, Live, Nonreplicating) for Preexposure Vaccination of Persons at Risk for Occupational Exposure to Orthopoxviruses: Recommendations of the Advisory Committee on Immunization Practices—United States, 2022

The above drugs and vaccines have proven to be effective at a certain level. However, recent interesting results have confirmed that they can cause significant adverse effects among humans [25]. Therefore, this question must be addressed, and there is a need for manufacturing and developing vaccines that target Mpox. The contraindication of ACAM2000 has been detailed in Fig. 3. Interestingly, Duchoslav et al. argued the implications of drug design and raised the concern that the use of many antiviral drugs traditionally only targets enzymes that include protease, polymerase and integrase, thus suggesting innovating strategies to combat Mpox by developing drugs that target an enzyme known as poxin.

## Prevention and control of Mpox

Devising standard prevention control measures such as contact tracing is key to combat the spread of infectious agents, such as in the case of Mpox virus. It aims to interrupt the transmission of infections by early detection of cases, identification of their data, including names and contact information of contacts considered a higher-risk group, places they have visited and advising them to closely monitor themselves for at least 21 days. Health workers in contact with confirmed or suspected cases must take all necessary preventive measures and ensure hygienic working conditions [12]. Equally important, contact tracing should focus on vulnerable populations with unsafe mobility potentially spreading the virus [26].

Ring vaccination, a valuable strategy for eradicating infection, can be effective if infectious cases are quickly identified. The success of this strategy depends on early immunization of close contacts of confirmed cases within 1–4 days between exposure and vaccination. This method, which was initially used to eradicate smallpox, has been effective in the recent Ebola outbreak eradication and in detecting vaccine efficacy [27]. Although this method is useful in assessing the efficacy of the vaccine and decreasing the spread of disease, it faces challenges in the context of Mpox. Those challenges rely on the fact that the virus affects mostly the gbMSM community who are stigmatized, thus making the needed testing and contact tracing essential for ring vaccination difficult [28].

Mpox, a zoonotic disease, spreads through animals, particularly in cases where humans are in close contact with animals, such as domesticated and farm animals. While this can be prevented, the possibility of reverse zoonosis has seldom been addressed. Reverse zoonosis, defined as human-to-animal transmission [28], can impede curbing disease transmission in a zoonotic context. This raises the question of whether necessary precautions have been implemented for infected humans to avoid transmitting the disease to animals to break the vicious cycle of transmission.

Another essential method to control Mpox is wastewater-based surveillance (WBS) to monitor or detect emerging water-borne pathogens. WBS has been crucial in polio epidemic eradication and has been effectively used during the coronavirus disease 2019 (COVID-19) pandemic [29]. Furthermore, WBS’ role in monitoring pathogens, such as adenovirus [30], enterovirus [31], rotavirus [32], dengue virus [33] and JC polyomavirus [34], has been documented and validated. Wastewater is considered an essential source for detecting communicable pathogens as it contains most biological materials such as skin, crusts, urine, faeces, semen, saliva, and nasal and respiratory secretions from all infected people, pre- and post-symptomatic [35]. Interestingly, WBS revealed the presence of the infection even before finding cases among individuals in the community [36]. Its ability to detect the Mpox virus and monitor its cycle in the community is essential. However, financial issues, lack of skills and substantial logistics render the method extremely difficult to implement in many countries.

## Recommendations

The 2022 Mpox outbreak has been a serious threat to public health, indicating the critical need to devise preventive measures necessary to keep the spread of Mpox under better control. This could be achieved by raising public awareness of the disease, isolating infected patients and providing healthcare providers with sufficient and necessary protective equipment such as masks, gloves and protective clothing. Vulnerable populations, as well as veterinarians, laboratory workers and healthcare workers who treat patients or animals with Mpox, must be vaccinated. Veterinarians are essential in controlling this outbreak since animals serve as reservoirs for Mpox. As a result, implementing screening programs for animals, especially those imported from severely impacted areas, can effectively stop the virus from spreading from animals to humans by setting up immunization programs for these infected animals. Equally important, healthcare workers must gain more knowledge about the clinical characteristics of Mpox and take precautions to avoid infection because any infection among healthcare personnel will lead the healthcare system to become overburdened. In similar previous conditions to this outbreak, seminars and learning workshops could be valuable for healthcare workers’ healthcare.

The screening program can also be beneficial in epidemic areas. Hence, any positive case must be treated with immediate effects and access to polymerase chain reaction (PCR)-ready must be given to healthcare facilities. This highlights the importance of treatment availability worldwide to treat suspected patients with Mpox with special precautions and standard infection control measures. Furthermore, patients should be in isolation during the duration of the disease, which is 2–4 weeks or until there are no more signs of the disease, such as lack of temperature for 72 h and lack of new lesions in the previous 2 days, and should follow protective measures such as using gloves, masks and covering unmasked lesions. Those preventive measures also include following basic hygiene measures, such as avoiding close contact with suspected individuals, practising safe hand washing, and sterilizing items, linens for example, that come in contact with suspected or confirmed cases. Equally important is the post-exposure prophylaxis. Persons in contact with a Mpox case should be vaccinated within 4 days of exposure and a fortnight if there are no signs of the disease, thus countering the onset of Mpox. It is therefore critical to ensure that the vaccines and drugs developed are safe and effective. Care should be exercised when handling personal use items of infected individuals, as the specific timeframe for the survival of viral particles on surfaces is unknown. In this line, waste management such as storage, disposal and handling of dressings and contaminated protective equipment of patients must be implemented on the basis of the Hazardous Materials Regulations (HMR) of the US Department of Transportation.

Most importantly, the gbMSM community should be sufficiently educated regarding self-protective measures such as patience or avoiding sex with affected or suspected individuals to have contracted the disease, reducing sex partners and use of condoms. Taking into consideration the vulnerability of gbMSM to Mpox, treatment and vaccination programs should first target this most affected community while assuring post-exposure prevention among those with multiple sex partners engaged in certain sexual activities. It is worth noting that the stigma faced by gbSMSM can impede tackling the infection, highlighting the need to destigmatize gbMSM. Avoiding this discrimination and stigmatization will require community-based consultation and action with gbSMSM to balance public health interventions with respect for individual privacy and confidentiality. This implies the duty of confidentiality that patients’ information should not be shared without patients’ consent or unless there is a legal requirement. Promoting ethical surveillance must address the risk of discrimination or stigmatization, thus providing equitable and accessible healthcare services.

## Conclusion

The recent multi-country Mpox outbreak of 2022 has presented several unique challenges and unanswered questions for the scientific and medical communities. The virus has been transmitted beyond its endemic regions in Central and West Africa, affecting several countries around the world. The emergence of atypical clinical presentations, particularly among gbMSM, has raised questions about the mode of transmission and the possibility of sexual transmission. The virus has also shown a raised severity in the current outbreak compared with previous ones, leading to increased cases and complications. Therefore, prevention and control strategies for Mpox should include contact tracing, ring vaccination and implementing comprehensive surveillance systems effective for early detection and containment of outbreaks, breaking the chain of transmission. Prevention also requires prompt action to screen humans and their related animals to tackle the possibility of reverse zoonosis. Considering the lessons learned from the COVID-19 pandemic, it is essential to apply the knowledge and experiences gained to manage and control the Mpox outbreak effectively. Addressing the unanswered questions surrounding Mpox transmission, developing effective vaccines and therapies, implementing robust prevention and control strategies and providing gbSMSM community-based consultation supported by ethical surveillance is crucial for mitigating the impact of the 2022 Mpox outbreak and preventing future outbreaks. International collaboration, data sharing and timely communication among healthcare professionals and public health authorities are essential in addressing this global public health emergency.

## Data Availability

Not applicable.
